# Supplementary material for: Determinants and barriers in early tuberculosis treatment in children at a primary health care facility in Kampala, Uganda; A mixed methods study
Source: PLoS One. 2025 Apr 16;20(4):e0321620. doi: 10.1371/journal.pone.0321620 (PMC12002530; doi:10.1371/journal.pone.0321620)
Supplement: Attachment — Submitted filename: Response to reviwers.docx [file pone.0321620.s003.docx]

**The editor**

**Plos one,**

**15^th^ /02/2025**

**Re: Response to reviewer’s comments**

| **S/No** | **Reviewer comment** | **Response** |
| --- | --- | --- |
|  | **Abstract** Capitalize the first letter when writing world health organization. It is not clear how diagnosis of TB in children less than 15 years is a barrier to ending TB. Is it the delay, under-diagnosis, misdiagnosis, etc? Methods: What was the study design? Mixed methods refer to methods of data collection (quantitative and qualitative) and not a study design. The methods section needs to be reorganized to reflect the key aspects of design, study size, sample size, data collection and analysis. Currently, the authors present methods for quantitative data collection, then analysis and back to qualitative data collection. Regarding the stated period of 1st-28th Feb 2023, it is not clear whether this refers to the time during which data was collected or that the participants were those that were initiated on TB treatment during this time. In the manuscript main text, the average number of patients with TB per month is indicated as 80. Results IQR refers to Interquartile range and therefore should be presented as a range rather than a single figure. How does referral of children less than 5 years to a national referral hospital become a barrier? It is delayed referral, delays in assessment at the national referral hospital or caregivers delaying going when referred? The loss of community contacts as a barrier is also not clear. | The first letter of world health organization has been capitalized.  Childhood TB is pauci bacillary in nature. This makes it particularly difficult to diagnose, contributing to treatment delays. This has been made clear in the abstract.  The study design has been revised to retrospective cohort mixed methods study.  Sample size, data collection and analysis methods have all been revised.  The authors appreciate the reviewer’s insight on this subject.  The study included **children under 15 years** who were treated for TB at Kisenyi Health Centre IV between 1**st February 2021 to 28th July 2023**. Data was collected between **7th and 21st July 2023. This has been made more clear under study population and data collection.**  Yes, IQR range is indeed a range and it has been revised as guided (30,80.9)  The referral system posed logistical and financial burdens to the caregivers of these children and this resulted into delays in treatment initiation.  With regards to loss of community contacts, caregivers would not bring childhood contacts of adults with TB to the hospital for early disease detection leading to late disease presentation.  These have been clarified under **qualitative results** |
|  | Introduction Paragraph 3: The 1st sentence is too long and the second part regarding post tuberculosis lung disease is unclear. Reference 8 should be about a paper describing the natural history on TB as stated. However, this very reference is a review paper discussing highlights of the Global TB report 2020 and not a research article. The manuscript is about time to initiation of TB treatment and related determinants and barriers. The introduction section should therefore focus on these 3 aspects. I note that there is no literature presented on determinants and barriers. The authors provide literature on delays to initiation of TB treatment with reference to studies among adults. Are there no studies in children which would be relevant to the study? If Yes, this needs to come out clearly. The factors at play in the different age groups are likely to be different. | The introduction has been revised as guided. The first paragraph gives the global disease burden followed by local disease burden.  The said reference has been replaced with the actual WHO annual TB report. The author thanks the reviewers for this observation.  The introduction has been further refined to focus on time to initiation, determinants and barriers as guided.  The limitations in children’s literature on the subject matter have been clearly stated. |
|  | Study design and setting The ‘mixed methods’ indicates that the study had both quantitative and qualitative data collection methods, but it is not a study design. Clarify if this was a cross sectional, cohort, case-control or any other study design. More details on the services. For example, what diagnostic tests for TB are available? To what extend are they accessible? What is the common practice for identifying and initiating TB treatment? Give more information about the 9 HWs that run the clinic. Cadre, skills set etc. What kind of patients attend the health centre given that it is in the city? Any referrals from other facilities to the TB clinic? Regarding the number of patients with TB seen per month, this is a relatively high number (20 per week). It is imperative that you describe the type of patients attending this clinic, where they come from. For example, are they from a known TB hotspot? | The study design has been revised to retrospective cohort mixed methods  The additional details with regards to services at the TB clinic have been added as advised under study design and setting.  More information is provided about the HW workers interviewed for the qualitative study under qualitative results  Kisenyi health center also serves informal settlements contributing to the high number of TB patients.  This has been explained very well under study setting. |
|  | Study population This is blank. Describe the study population. Add a sub-title for procedures or data collection. Then you can proceed to describe how the quantitative and qualitative data was collected. The study methods for the quantitative part of the study are confusing. The first paragraph indicates that data was extracted from the registers and then later on, enrolment of study participants is mentioned. Provide more information about the TB register. What kind of information is recorded and with what level of accuracy? For example, one of the study objectives was to determine the time from start of symptoms to initiation of treatment. Does the register capture such information? The authors need to provide detailed and clear information on the methods including study design, setting, study population, eligibility criteria, sample size estimation and sampling process for both the qualitative and quantitative parts of the study. The data collection procedures need to be well described, right from identifying potential participants/screening, consent and assent and tools used to collect the data, such as questionnaires, etc. I note that some of the data collection processes for the qualitative part are described under data management. This information should be transferred to the right section | Study population, sampling procedure and sample size sub sections have been added as you have advised.  The collection of quantitative data was through data extraction from the TB register. This has been made clear under sampling procedure.  The TB register has been well described under a section of study variables. We again thank the reviewers for this insight.  Quantitative and qualitative sections have been re organized to make it easier on the reader.  The inclusion and exclusion has been clearly stated under the study populations. |
|  | Data management and analysis This section should be presented reflecting the objectives of the study and the corresponding method used to manage and analyse data. The authors need to clearly describe how the data for the different objectives was analysed. a) Median time to initiation of treatment b) Determinants of initiation of treatment c) Barriers to timely initiation of treatment Ideally, when analysing any data set, one would first explore the data to check whether it is normally distributed or skewed and decide on whether to present as average (for normally distributed data) or median and IQR for skewed data. In this study, the 1st objective was very specific on median time to initiation of treatment. Was this based on the assumption that the data would be skewed? Kaplan Mier survival curves were used to analyse the time to initiation of treatment. Please present the results of the graphs in the main text or as supplementary material for review and validation. | The analysis plan has been revised as you have guided.  The analysis plan for the descriptive statistics has been added.  Kaplan Meir curves were used to estimate median time to treatment initiation and cox proportional hazards regression was used for assessing determinants of time to initiation, Thematic analysis was used to summarize qualitative data.  This is all presented under data management and analysis. |
|  | Results The flow chart needs to be improved by providing details on the missing data. What is the outcome variable that was noted to be missing? Provide details on the independent variables that were missing and the corresponding number of participants in which they were missing. Table 2 is missing. Table 3 and 4 can be combined in one table The results from the qualitative part of the study were based on what the health workers think and not necessary the truth and should be discussed as such. | More details have been added on the flow chart clarify exactly what variables had missing information  Tables have been merged as advised |
|  | Discussion The terms time to initiation, median time, time interval are used interchangeably in the first paragraph but mean different things. The authors provide information that compares the results with studies for other parts of the world. This is good. However, how does the results compare with the WHO recommendation and what are the implications?  Reviewer #2: The introduction is very short. The importance and necessity of the study should be fully explained. The discussion to be completed in based on the relevant articles. The findings to be completed in based on the purpose of the study. The results of the study do not expand the boundaries of science. Sources should be edited according to reference writing guidelines and journal format. The strengths and limitations of the study to be mentioned. It is suggested to remove the references before 2015 and replace them with new ones. | The discussion has been revised as guided by the reviewers.  Recommendations on key research findings are within the text of the discussion.  The introduction has been expounded further to highlight importance and necessity of the study.  The limitations of the study have stated as advised |
